# Micellization Studies of Block Copolymers of Poly(N-vinyl pyrrolidone) and n-Alkyl-Substituted Poly(vinyl esters) in Tetrahydrofuran

**DOI:** 10.3390/polym17212842

**Published:** 2025-10-24

**Authors:** Nikoletta Roka, Vasileios-Christos Skiadas, Areti Kolovou, Theodosia-Panagiota Papazoglou, Marinos Pitsikalis

**Affiliations:** Industrial Chemistry Laboratory, Department of Chemistry, National and Kapodistrian University of Athens, Panepistimiopolis Zografou, 15771 Athens, Greece; bill.skd@hotmail.com (V.-C.S.); aretikolovou.chem@gmail.com (A.K.); pnpapazoglou@gmail.com (T.-P.P.); pitsikalis@chem.uoa.gr (M.P.)

**Keywords:** poly(N-vinyl pyrrolidone) (NVP), poly(vinyl esters) (VEs), amphiphilic block copolymers, micelles, light scattering

## Abstract

The association behavior of amphiphilic block copolymers of N-vinyl pyrrolidone (NVP) and several vinyl esters (Ves) (PNVP-b-PVEs), as exemplified by vinyl butyrate (VBu), vinyl decanoate (VDc), and vinyl stearate (VSt), was studied in tetrahydrofuran (THF), which serves as the selective solvent for the PVE blocks. Static (SLS) and dynamic light scattering (DLS) techniques were adopted as the tools to investigate micellar properties and acquire information regarding the degree of association, the hydrodynamic radii, and the shape of the aggregates. In addition, CONTIN analysis provided insights concerning the association equilibria in THF solutions. The effect of the chemical structure of the corona-forming PVE block on the association process was investigated. Finally, the experimental results were compared with those obtained in previous studies describing the micellization properties of block copolymers consisting of PNVP and polymethacrylate blocks in the same selective solvent.

## 1. Introduction

The most fascinating class of polymeric materials is without any doubt the family of block copolymers [1,2]. This is attributed to the following main reasons: (a) Block copolymers consist of two or more distinct polymer segments covalently linked in a linear sequence. This unique structure allows them to exhibit diverse characteristics, which are contingent upon the specific polymers used and their arrangement [3,4,5]. (b) There are numerous combinations of different blocks that can be combined in one structure, such as hydrophilic, hydrophobic, amorphous, crystalline, flexible, rigid materials, etc. [6,7]. (c) Well-defined copolymers with narrow molecular weight distributions and low chemical heterogeneity can be synthesized through a huge variety of polymerization techniques and combinations such as anionic, cationic, controlled radical, ring opening, ring opening metathesis, and coordination polymerization [8,9,10,11,12,13,14,15,16,17,18,19,20,21,22,23,24]. (d) Block copolymers employ their unique characteristic to promote self-assembly. This means that they are organized as supramolecular structures either in the absence of any solvent or in solvents good for one of the blocks [25,26]. In the absence of any solvent, the self-organization process produces specific microstructures, such as cylindrical, spherical, lamellar and cubic microphases, etc. [27,28], whereas in solvents selective for one of the blocks to the formation of micellar structures [29,30,31,32,33], the micelles usually adopt the core–corona structure [34,35,36]. The non-soluble block forms the core, whereas the surrounding protective soluble block forms the corona of the supramolecular assemblies.

Amphiphilic block copolymers are a special subclass of copolymers bearing one block that is water-soluble and another one that is insoluble in water [37,38,39,40,41,42,43]. The characteristic of these structures is that they offer the possibility to self-assemble both in water and in suitable organic solvents. For this reason, countless applications have appeared in the literature based on these materials, including the fields of drug encapsulation and delivery, the biomedical sector (nanocarriers for gene therapy, stimuli-responsive drug carriers, biodegradable hydrogels, etc.), coating and surface modification (antifouling coatings, self-healing applications, etc.), nanotechnology and electronics (nanoreactors, nanocatalysis, organic electronics, flexible displays, etc.), cosmetics and personal care products (surfactants, emulsifiers, encapsulation of active ingredients, etc.), and water purification systems, etc., [39,44,45,46,47,48,49,50,51,52,53].

In previous studies, the preparation of various amphiphilic statistical and block copolymers has been reported based on poly(N-vinyl pyrrolidone) (PNVP) as the hydrophilic block and several polymethacrylates and poly(vinyl ethers) (PVEths) as the water-insoluble blocks, and their micellization behavior was examined both in specific organic solvents and in water [5,54,55,56,57,58,59,60,61,62]. In the frame of this project, diblock copolymers consisting of PNVP and poly(vinyl esters) (PVEs) with alkyl side groups were synthesized and thoroughly characterized [63]. More specifically, the synthesis of block copolymers (PNVP-b-PVEs) with poly(vinyl butyrate) (PVBu) poly(vinyl decanoate) (PVDc), and poly(vinyl stearate) (PVSt) blocks was reported by employing Reversible Addition Fragmentation Chain Transfer (RAFT) approaches.

Previous efforts have been documented for the preparation of statistical and block copolymers of various PVEs. Most of them were based on poly(vinyl acetate), PVAc [64,65,66]. However, other VE monomers have been employed as well, including vinyl pivalate, vinyl benzoate, and vinyl octanoate [67,68,69,70,71]. The most frequently mentioned system in the literature is that of PNVP-b-PVAc block copolymers. The main target of these works was the study of the micellar properties of these copolymers in aqueous solutions and their ability to encapsulate hydrophobic compounds and perform as potential drug delivery systems. On the other hand, only a few works describing the synthesis of diblocks between NVP and other VEs have been previously published in the literature, and in addition no effort was given to provide results regarding the micellization properties of these diblocks in organic solvents that are good solvents exclusively for the PVE blocks.

In order to fill this gap in the literature, the self-assembly behavior of PNVP-b-PVBu, PNVP-b-PVDc, and PNVP-b-PVSt was studied in tetrahydrofuran (THF), which is a selective solvent for the PVE blocks and a non-solvent for the PNVP blocks [72].

## 2. Materials and Methods

The values of the refractive index increments, dn/dc, at 25 °C were determined using a Chromatix KMX-16 refractometer (Milton Roy, LDC Division, Riviera Beach, FL, USA), which operates at 633 nm and was calibrated with solutions of sodium chloride in water.

Dynamic light scattering, DLS, experiments were carried out on a BI-200SM goniometer system (Brookhaven Instruments, Holtsville, NY, USA), which is equipped with a 40 mW laser at a wavelength of 640 nm. Correlation functions were analyzed using both the cumulant method and the CONTIN software package (Brookhaven Instruments, Holtsville, NY, USA) [73]. Data were collected at scattering angles of 45°, 90°, and 135° at a temperature of 25 °C. For DLS measurements, five replicates were performed for each concentration, whereas every sample was measured twice, starting from different initial concentrations.

In all micellar systems studied, the angular dependence of the Γ/q^2^ ratio (Γ denotes the correlation function’s decay rate, and q denotes the scattering vector) was negligible. Therefore, the values of the apparent translational diffusion coefficients at infinite dilution, D_o,app_, were extracted using Equation (1):

D_app_ = D_0, app_(1 + kDc) (1)

The corresponding apparent hydrodynamic radii, R_h_, were calculated by employing the relation of Stokes–Einstein (Equation (2)):

R_h_ = kT/6πη_s_D_0, app _ (2)

with k being the Boltzmann’s constant and T being the absolute temperature, whereas η_s_ is the solvent viscosity.

Static light scattering, SLS, experiments were conducted using the same BI-200SM system used for the DLS measurements, and data were processed using the Zimm plot module of the Particle Explorer software (v1.2.0.6868, Brookhaven Instruments, Holtsville, NY, USA). The data was collected at 25 °C at the scattering angles of 45°, 60°, 75°, 90°, 105°, 120°, and 135°. SLS measurements were conducted in ten replicates for each concentration. Again, every sample was measured twice, starting from different initial concentrations.

For all the measurements, initially a stock solution was prepared by direct dissolution of the respective sample in THF. The solvent was previously dried overnight in sodium and distilled just prior to its employment. The stock solutions were left overnight at room temperature, and then gently heated in an oven at 40 °C for at least 3 h, thus facilitating the dissolution of the polymers and obtaining micellar structures in equilibrium. The stock solutions were further diluted with dry THF to obtain lower-concentration solutions. The range of concentrations for all the samples was between 5 × 10^−3^ and up to 7 × 10^−2^ g/mL for both SLS and DLS measurements. Although all samples appeared crystal-clear without turbidity or insoluble particles, they were filtered through 0.22 μm pore size hydrophobic PTFE filters (Millex-LCR, Millipore, Cork, Ireland) to ensure the removal of any dust before being directly introduced into the scattering cell in the order of increasing concentration.

Viscometry measurements of dilute solutions were conducted at 25 °C using Cannon-Ubbelohde dilution viscometers (State College, PA, USA) with a Scott-Geräte AVS 410 automatic flow timer (Heidelberg, Germany). Viscometric data were analyzed by employing both the Huggins equation

η_sp_/c = [η] + k_H_[η]^2^c + … (3)

and the Kraemer equation

lnη_r_/c = [η] + k_K_[η]^2^c + … (4)

where η_r_, η_sp_, and [η] are the relative, specific, and intrinsic viscosities, respectively. Moreover, k_H_ and k_K_ are the Huggins and Kraemer constants, respectively. Viscometric radii, R_v_, were calculated from the equation

R_v_ = (3/10πN_A_)^1/3^([η] M_w,app_)^1/3^ (5)

where M_w,app_ is the average molecular weight determined by light scattering measurements.

Transmission electron microscopy (TEM) experiments were carried out using a Hitachi HT7800. Carbon-coated copper TEM grids with a size of 200 mesh (ProSciTech Pty. Ltd., Thuringowa, Australia) were employed. An aliquot (5 μL) of the sample was pipetted onto each grid. The solution was allowed to be adsorbed for 1 min. The grids were then manually blotted using Whatman 541 filter paper to remove the excess liquid on the grids. The grids were then left to air-dry at room temperature for approximately 10 min. No staining protocol was applied to the grid preparation for all samples.

## 3. Results and Discussion

### 3.1. Preparation of the Block Copolymers of NVP and VEs

The synthesis of the PNVP-b-PVEs was described in detail in a previous publication [63]. Very briefly, the polymerization of NVP was initially carried out using O-ethyl S-(phthalimidylmethyl) xanthate, which served as the CTA. The produced polymer was isolated, purified, characterized, and further used as the macro-CTA for the subsequent polymerization of the respective VE monomer, enabling the formation of well-defined block copolymers. Table 1 summarizes the molecular characteristics of these blocks (average molecular weights, M_n_, and dispersity values, Ð).

### 3.2. Self-Assembly Behavior of the PNVP-b-PVE Block Copolymers in THF Solutions by Static Light Scattering

**Figure 1:**
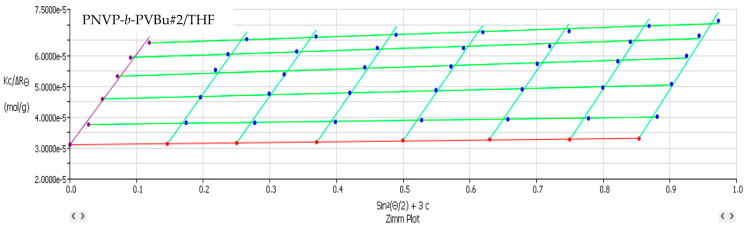
Zimm plot (Kc/ΔR_θ_ vs. sin^2^(θ/2)) corresponding to PNVP-b-PVBu #2 in THF.

**Figure 2:**
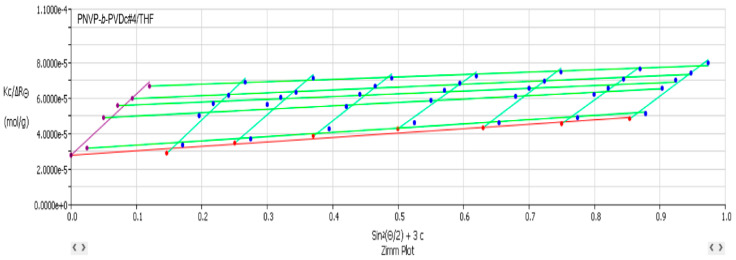
Zimm plot (Kc/ΔR_θ_ vs. sin^2^(θ/2)) corresponding to PNVP-b-PVDc #4 in THF.

**Figure 3:**
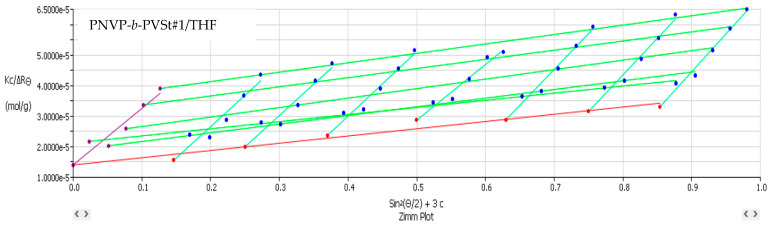
Zimm plot (Kc/ΔR_θ_ vs. sin^2^(θ/2)) corresponding to PNVP-b-PVSt #1 in THF.

Table 2 lists the SLS data from the PNVP-b-PVEs block copolymers in THF, whereas characteristic Zimm graphs from these measurements are presented in Figure 1, Figure 2 and Figure 3.

It is of paramount importance to verify that the micellar solutions are in equilibrium when performing light scattering measurements. Heating the initial stock solution for several hours is usually enough to promote equilibrium structures, especially when the supramolecular structures are not very large, as seen in this case. Verification was made by employing measurements starting with stock solutions of different initial concentrations. If the solutions are not under equilibrium, the different stock solutions would provide different results regarding the degree of association and the hydrodynamic radius. In our case all the results were comparable, confirming that the samples are in thermodynamic equilibrium.

In previous studies examining the self-assembly properties of statistical and block copolymers of PNVP with polymethacrylates, it was concluded that THF is not able to promote the formation of large multimolecular micelles [54,61]. The weight-average degrees of association, N_w_, were higher in the case of the block copolymers [55]. Nevertheless, even in this case, the N_w_ values were not very high (N_w_ < 10). These results look reasonable for the statistical copolymers due to the specific distribution of the individual monomer units along the polymer backbone. However, for the block copolymers with two distinct constituent chains connected with a covalent bond, it would be expected that a more pronounced association behavior could be feasible. The main conclusion from the previous systems is that unimolecular micelles are primarily produced from statistical copolymers, in contrast to the block copolymers, which form compact micelles with a low aggregation number in THF solutions.

This behavior was more or less confirmed in the present study, which studies the self-organization process of the PNVP-b-PVE block copolymers in THF. The experimental data from the SLS measurements revealed low aggregation numbers in almost all cases. This can be attributed to the comparatively reduced molecular weights of the samples. However, the main reason is correlated with the low ability of THF to promote the formation of large aggregates. Slightly higher N_w_ values were measured for the PNV-b-PVSt copolymers, indicating that the long alkyl side chain of the corona-forming block, PVSt, through hydrophobic interactions, further stabilizes the corona of the supramolecular structures or even facilitates intermicellar interactions, leading to more complex patterns of association.

It has to be mentioned that the copolymers containing the greatest proportion of PNVP have slightly lower N_w_ values, meaning that compact crew-cut star-like micelles prevail in THF solutions. The surrounding corona of the supramolecular structures with the extended side alkyl chains of the respective PVEs is able to stabilize lower aggregation numbers. In addition, the reduced values of the second virial coefficient, A_2_, from SLS measurements in THF solutions verify the existence of micelles with unimolecular characteristics or a low aggregation number. A similar behavior has been observed in the case of statistical and block copolymers of PNVP and polymethacrylates.

### 3.3. Self-Assembly Behavior of the PNVP-b-PVE Block Copolymers in THF Solutions by Dynamic Light Scattering

**Figure 4:**
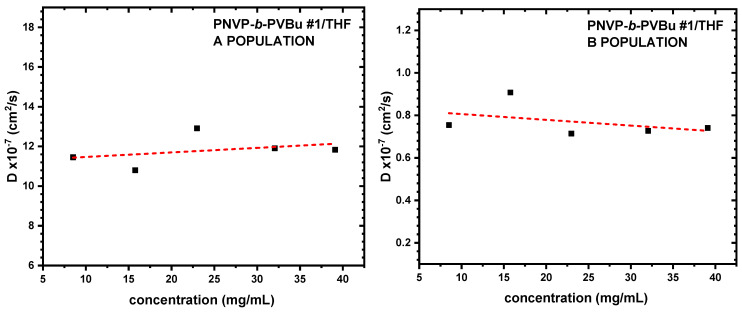
D vs. c plots of PNVP-b-PVBu #1 in THF.

**Figure 5:**
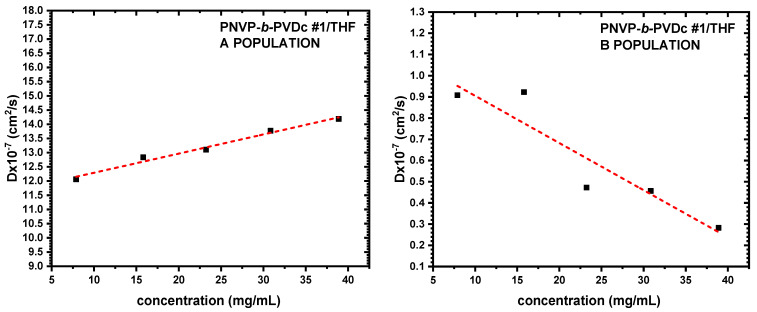
D vs. c plots of PNVP-b-PVDc #1 in THF.

**Figure 6:**
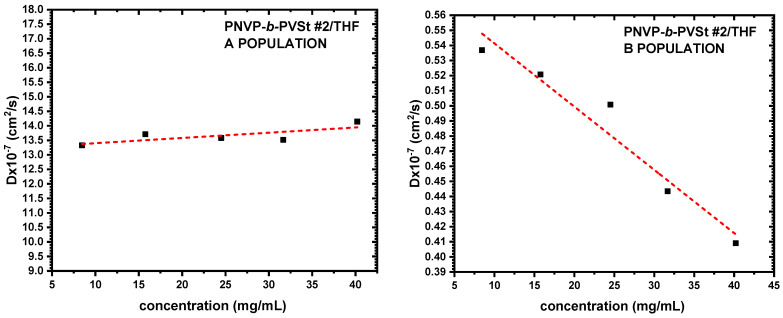
D vs. c plots of PNVP-b-PVSt #2 in THF.

The findings obtained from SLS measurements were additionally validated and elucidated by the DLS results. CONTIN analysis is able to offer distribution analysis of the various populations that may be present in the selective solvent. Therefore, DLS data provide deeper insight into the micellization process. Table 3 summarizes the specific data, while characteristic DLS plots from the various copolymer families are shown in Figure 4, Figure 5 and Figure 6.

**Figure 7:**
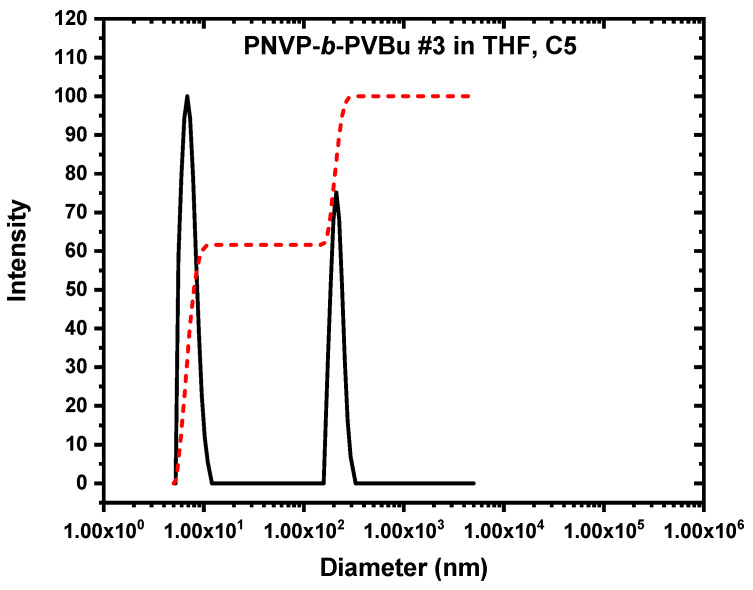
CONTIN plots (black line scattering intensity vs. diameter) of PNVP-b-PVBu #3 (c = 3.953 × 10^−2^ g/mL). The red line is the integration line of the two peaks.

As expected, the DLS measurements confirm and further fortify the corresponding SLS data. Regarding the PNVP-b-PVBu copolymer, CONTIN analysis revealed the presence of two distinct populations in the THF solutions. Characteristic CONTIN plots for sample PNVP-b-PVBu #3 are given in Figure 7. Judging from their relative size, it can be concluded that there is an equilibrium between unimolecular and typical core–shell multimolecular micelles. The unimolecular micelles dominate in the selective solvent, since the high-R_h_-value population ranges between 25 and 50% in all concentrations and for all samples. Taking into account that larger particles provide higher scattering intensity values than the small ones, it can be derived that the contribution of the multimolecular micelles is much less than 20% in the solution. This finding aligns with the reduced degrees of association measured by SLS measurements.

The k_D_ levels in THF were low, as was expected from the modest A_2_ values from the SLS data. This outcome is justifiable given the established relationship between k_D_ and A_2_, which is defined by the equation below.

k_D_ = 2A_2_M − k_f_ − u (6)

In this equation M represents the molecular weight, k_f_ denotes the coefficient related to the concentration dependence of the friction coefficient, and u stands for the partial specific volume of the polymer.

In addition to these observations, no angular dependence was obtained from the DLS measurements, confirming the formation of spherical structures. Judging from the values of the polydispersity factor μ_2_/Γ^2^, with μ_2_ being the second moment calculated via cumulant analysis and Γ being the correlation function’s decay rate, these structures were relatively polydisperse. These values were higher than 0.1 for all samples.

**Figure 8:**
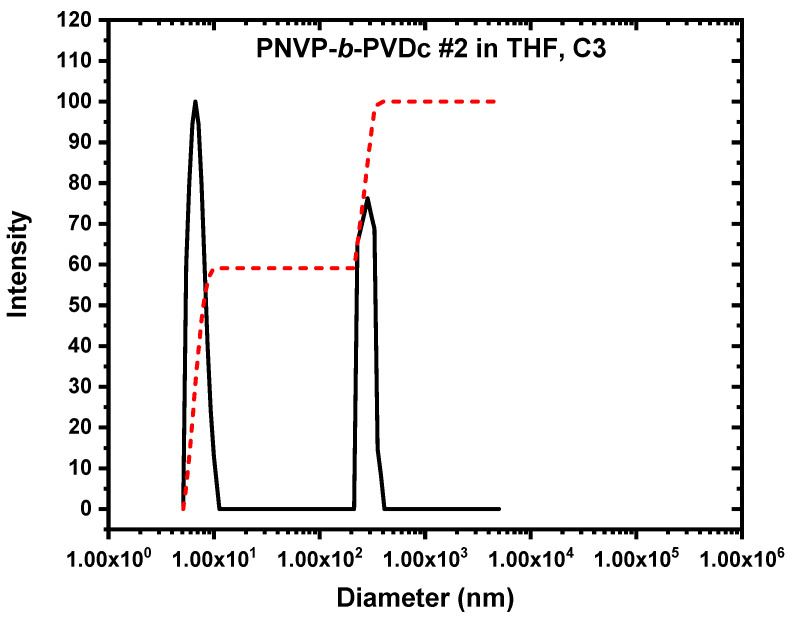
CONTIN plots (black line scattering intensity vs. diameter) of PNVP-b-PVDc #2 (c = 2.436 × 10^−2^ g/mL). The red line is the integration line of the two peaks.

The DLS results for PNVP-b-PVDc were comparable to those previously presented for the PNVP-b-PVBu samples. CONTIN plots verified the establishment of equilibrium between unimolecular and multimolecular micelles. An illustrative example is presented in Figure 8. The population of the core–shell micelles is in the same order as in the previous case (ranging between 20 and 50%); however, the R_h_ values of these structures are relatively lower compared to the multimolecular micelles coming from the PNVP-b-PVBu copolymers. This result indicates that the micelles with the corona from PVDc chains are more compact; this is attributable to the stronger hydrophobic interactions of the alkyl substituents of the PVDc chains. Otherwise, spherical, relatively polydisperse structures were obtained in this case as well.

**Figure 9:**
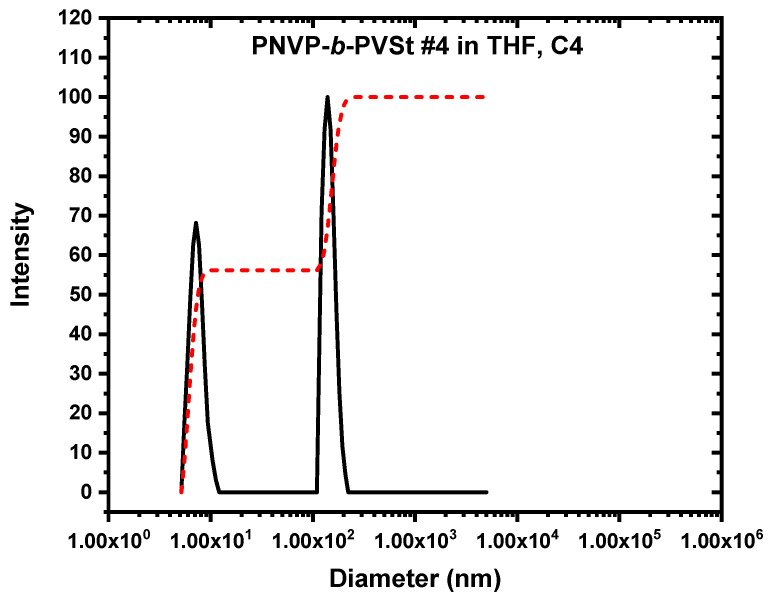
CONTIN plots (black line scattering intensity vs. diameter) of PNVP-b-PVSt #4 (c = 3.184 × 10*^−^*^2^ g/mL). The red line is the integration line of the two peaks.

The situation was slightly differentiated in regard to the PNVP-b-PVSt block copolymers. From the SLS data, it was concluded that the levels of association were relatively higher in comparison with the other two families of copolymers. The results from DLS and especially the CONTIN method demonstrated that the equilibrium is shifted in this case towards the micellar structures. Their population is typically more than 50% and up to 70% for specific copolymers and at higher concentrations. Of course, the real population, as explained earlier, is much lower; nevertheless, it is much higher than that of the other two types of block copolymers. The potential of stronger interactions among the PVSt chains, due to side chain crystallization, is responsible for this observation. Characteristic CONTIN plots are given in Figure 9. The second population is not only more pronounced in the overall content in the solution but also has considerably higher R_h_ values. Furthermore, the size of the second population is more uniform compared to the other two families of copolymers, indicating a similar process of organization. This is attributed to the high VSt content and the stronger hydrophobic interactions between the poly(vinyl ester) chains, which stabilize the multimolecular structures. Very low or even negative k_d_ values were measured across all cases, in accordance with the corresponding reduced A_2_ values reported in the SLS measurements and the more extended association phenomena reported for this copolymerization system.

Evaluating the self-assembly behavior in relation to other statistical and block copolymers of PNVP with polymethacrylates in the same selective solvent (THF) revealed similarities and differences. The degrees of associations are higher than those of the statistical copolymers, where unimolecular micelles almost exclusively dominate. However, compared to the respective block copolymers, the association behavior is more pronounced, especially with respect to the PNVP-b-PVSt copolymers, where a higher tendency for the formation of multimolecular micelles was obtained. The polymethacrylates, as solvophilic blocks, generate small and compact micellar structures, as revealed by CONTIN plots without equilibrium and with unimolecular micelles. Therefore, the nature of the solvophilic block dramatically affects the self-assembly process.

### 3.4. Self-Assembly Behavior of the PNVP-b-PVE Block Copolymers in THF Solutions by Dilute Solution Viscometry

**Figure 10:**
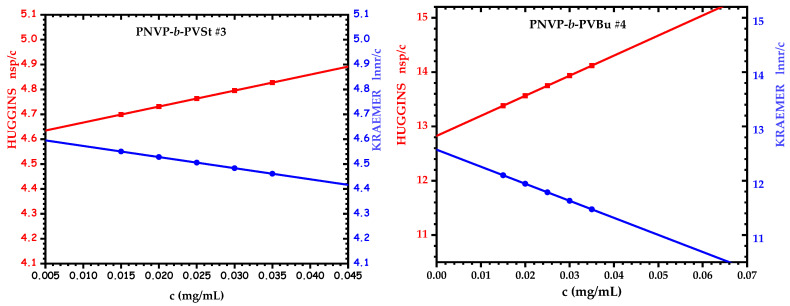
Viscometry plots of the dilute solutions for samples PNVP-b-PVBu#4 and PNVP-b-PVSt#3.

The viscometry data of the dilute solutions are given in Table 4, whereas characteristic plots from the measurements are shown in Figure 10.

The intrinsic viscosity values from both the Huggins and Kraemer approaches were in close agreement for all samples, confirming the validity of the methods. The K_H_ values were relatively high in most cases, although the intrinsic viscosity values were not very high. This result is a manifestation of the presence of strong hydrodynamic interactions in the THF solution, which occur as a result of the aggregation process. The R_v_ values are very close to the R_h_ values, as measured by DLS measurements corresponding to the first population in THF solutions. An equilibrium between unimolecular and multimolecular micelles is established according to the CONTIN analysis of the DLS data. The viscometry results of the dilute solutions confirm that the multimolecular micelles are not very stable and that they disassociate to the corresponding unimolecular micelles upon the existence of shear forces, which are developed in the capillary viscometer. This behavior has been previously reported in aggregating systems [74,75].

### 3.5. Self-Assembly Behavior of the PNVP-b-PVE Block Copolymers in THF Solutions by TEM Imaging

**Figure 11:**
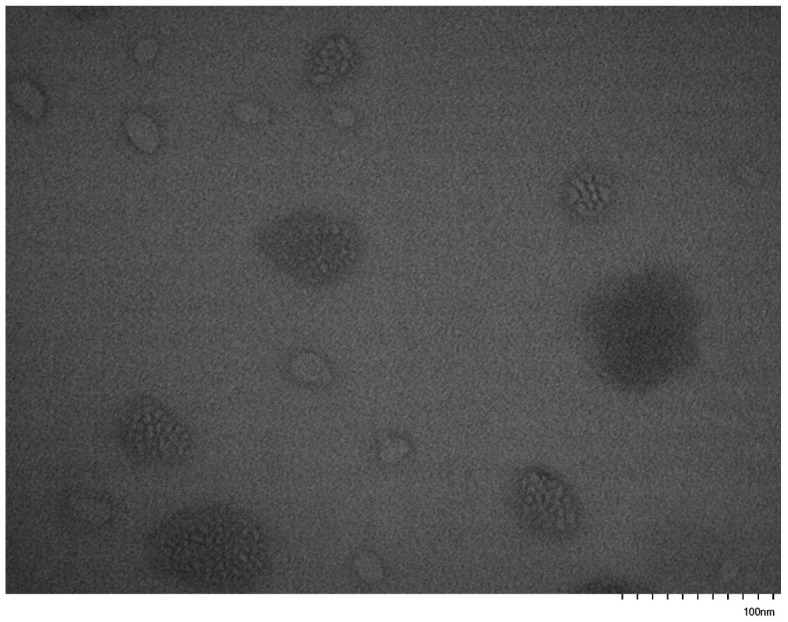
TEM image from sample PNVP-b-PVBu #3.

**Figure 12:**
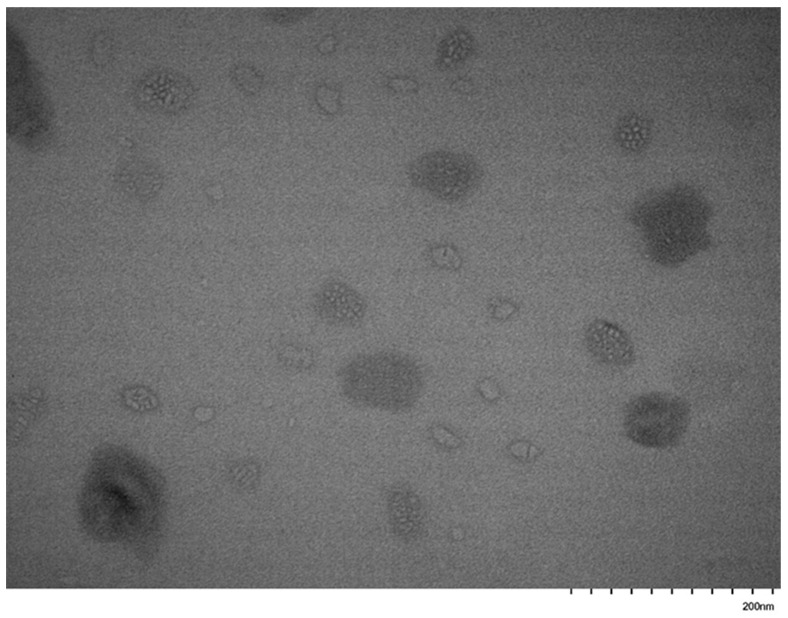
TEM image from sample PNVP-b-PVBu #2.

**Figure 13:**
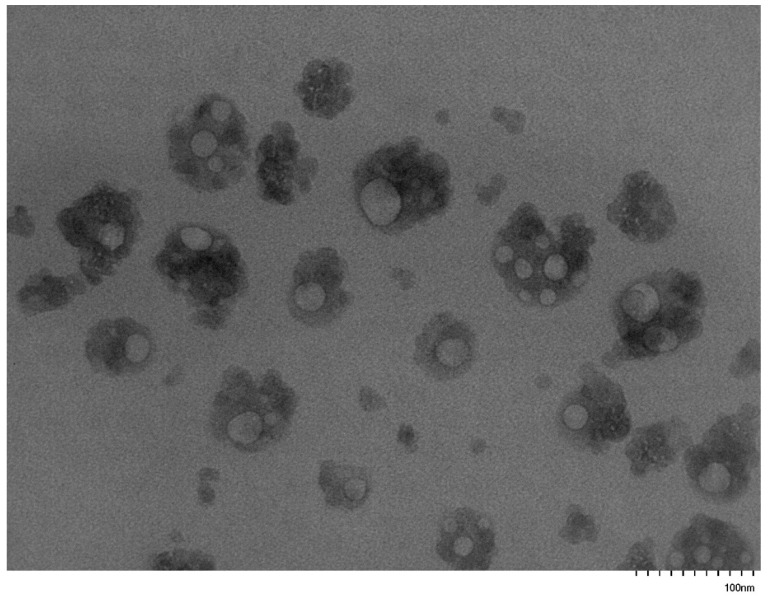
TEM image from sample PNVP-b-PVDc #2.

**Figure 14:**
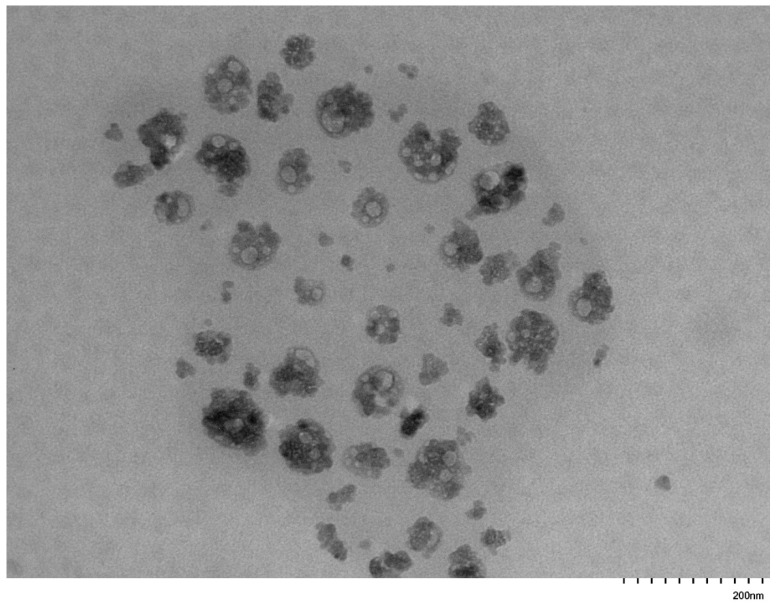
TEM image from sample PNVP-b-PVDc #4.

**Figure 15:**
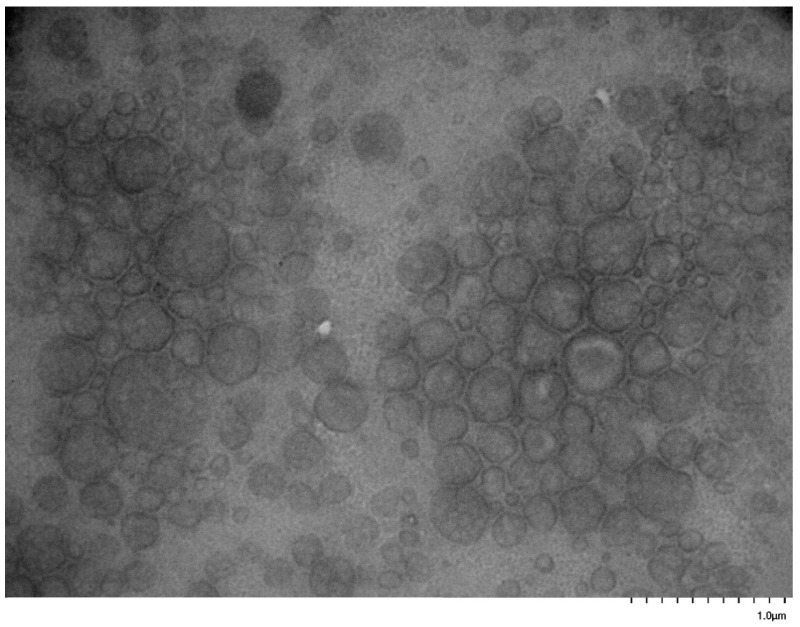
TEM image from sample PNVP-b-PVSt #1.

**Figure 16:**
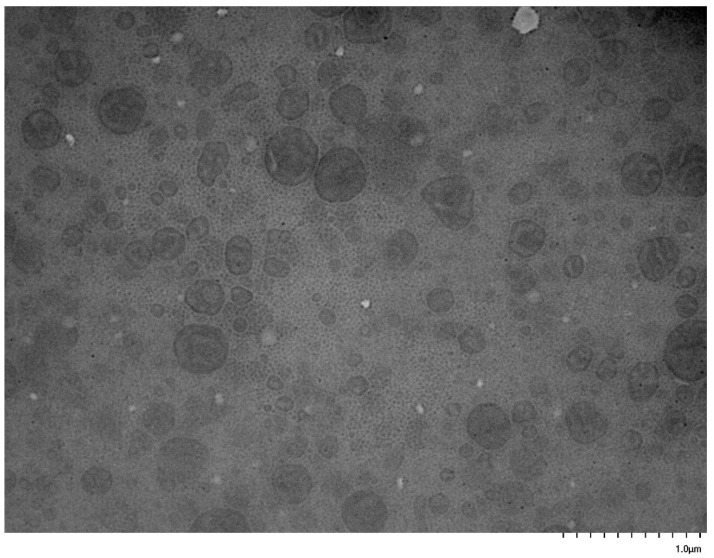
TEM image from sample PNVP-b-PVSt #2.

TEM images were collected on carbon-coated copper grids without performing staining. In an effort to enhance the contrast of the micellar structures and improve the quality of the TEM images, the grids were stained with uranyl acetate. However, this procedure was not efficient, since the strong interaction of the pyrrolidone rings with uranium resulted in a black surface without any possibility to identify supramolecular structures on the grids. Therefore, the images were obtained without staining. Characteristic images from the micellar solutions in THF are given in Figure 11, Figure 12, Figure 13, Figure 14, Figure 15 and Figure 16.

These figures confirm the results that have been drawn from the DLS measurements. It is obvious that there is an equilibrium between large multimolecular micellar structures and very small unimolecular micelles. The large structures are polydisperse and seem to be constructed as assemblies of monomolecular micelles. This conclusion can explain how easily these structures disassemble to unimolecular micelles upon the application of shear forces during the flow of the solutions with the supramolecular structures in the capillary tube of the viscometer. Finally, the radii of the observed micellar entities from the TEM images seem to be larger than those measured by DLS. This result is reasonable and commonly observed in the literature, since in TEM imaging, the structures are expanded on the surface of the grid in the absence of any solvent [76,77].

This work will be further expanded with further analysis of the micellar characteristics of these block copolymers in aqueous solutions.

## 4. Conclusions

The self-assembly properties of amphiphilic block copolymers bearing a hydrophilic poly(N-vinyl pyrrolidone) (PNVP) block and several hydrophobic poly(vinyl ester), (PVE) blocks (PNVP-b-PVEs), including poly(vinyl butyrate) (PVBu), poly(vinyl decanoate) (PVDc), and poly(vinyl stearate) (PVSt), were explored in tetrahydrofuran (THF), which is a selective solvent for the PVE blocks. The studies were based on static (SLS) and dynamic light scattering (DLS) measurements. Rather low degrees of aggregation were found by SLS data, indicating the presence of unimolecular or small micelles in THF. More details concerning the association equilibria in THF solutions were traced by the DLS techniques and the CONTIN analysis. In all cases, an equilibrium between unimolecular and compact and spherical multimolecular micelles is established in THF solutions. Upon increasing the size of the alkyl side group of the PVE blocks, the equilibrium is shifted towards the multimolecular structures. In all cases the supramolecular structures are relatively polydisperse. These experimental results are in agreement with previous studies reporting the micellization properties of block copolymers of PNVP with polymethacrylates in the same selective solvent. However, in the present system, the trend towards the formation of multimolecular micelles is more pronounced, as revealed by the CONTIN analysis. These conclusions were further conformed by dilute solution viscometry measurements and TEM images.

## Figures and Tables

**Table 1:** Molecular features of the block copolymers.

	Macro CTA (PNVP) ^a^		Block Copolymers ^a^		NVP	Vinyl Ester
Sample	M_n_ 10^3^ (Daltons)	Ð	M_n_ 10^3^ (Daltons)	Ð	% mol ^b^	% mol ^b^
PNVP-b-PVBu #1	8.5	1.30	16.0	1.90	22	78
PNVP-b-PVBu #2	8.9	1.35	15.5	1.54	48	52
PNVP-b-PVBu #3	8.9	1.35	17.5	1.40	57	43
PNVP-b-PVBu #4	28.0	1.27	32.0	1.32	84	16
PNVP-b-PVDc #1	5.5	1.47	12.5	1.60	38	62
PNVP-b-PVDc #2	8.5	1.30	11.0	1.45	56	44
PNVP-b-PVDc #3	8.5	1.30	12.5	1.31	63	37
PNVP-b-PVDc #4	9.5	1.36	10.5	1.36	93	7
PNVP-b-PVSt #1	7.5	1.30	10.4	1.51	61	39
PNVP-b-PVSt #2	8.5	1.30	10.5	1.44	78	22
PNVP-b-PVSt #3	8.1	1.30	12.5	1.22	83	17
PNVP-b-PVSt #4	8.1	1.30	10.9	1.37	85	15

a. by SEC in CHCl_3_. b. by ^1^H-NMR.

**Table 2:** SLS results of PNVP-b-PVE block copolymers in THF.

Sample	M_w_ × 10^−4^	N_w_	A_2_ × 10^4^ (cm^3^ mol/g^2^)
PNVP-b-PVBu #1	15.9	9.94	1.40
PNVP-b-PVBu #2	3.22	2.08	4.39
PNVP-b-PVBu #3	3.62	2.07	3.86
PNVP-b-PVBu #4	5.69	1.78	0.69
PNVP-b-PVDc #1	6.35	5.08	2.07
PNVP-b-PVDc #2	3.35	3.05	2.50
PNVP-b-PVDc #3	10.5	8.40	3.50
PNVP-b-PVDc #4	3.59	3.42	5.16
PNVP-b-PVSt #1	7.13	6.86	2.78
PNVP-b-PVSt #2	3.82	3.64	1.48
PNVP-b-PVSt #3	18.6	14.88	1.90
PNVP-b-PVSt #4	4.44	4.07	1.30

**Table 3:** Summary of DLS measurements for block copolymers in THF.

Sample	Do (cm^2^/s)	K_d_	R_ho_ A (nm)	R_ho_ B (nm)
PNVP-b-PVBu #1	11.2405	2.04	4.22	48.45
PNVP-b-PVBu #2	10.4839	4.63	4.53	32.97
PNVP-b-PVBu #3	9.9667	8.58	4.76	170.06
PNVP-b-PVBu #4	9.19953	−2.98	5.16	96.31
PNVP-b-PVDc #1	14.2491	0.96	3.33	35.82
PNVP-b-PVDc #2	11.6172	5.81	4.08	76.10
PNVP-b-PVDc #3	15.1746	−1.11	3.13	15.58
PNVP-b-PVDc #4	14.0547	0.29	3.38	85.11
PNVP-b-PVSt #1	13.2195	1.37	3.59	81.37
PNVP-b-PVSt #2	12.2076	3.09	3.89	71.94
PNVP-b-PVSt #3	13.8421	1.40	3.43	94.93
PNVP-b-PVSt #4	14.3697	0.32	3.30	84.08

**Table 4:** Viscometry results of the dilute solutions in THF.

Sample	[η]_H_ mL/g	[η]_K_ mL/g	K_H_	R_v_, nm
PNVP-b-PVBu #1	12.30	11.92	0.33	6.76
PNVP-b-PVBu #4	12.82	12.57	0.43	4.87
PNVP-b-PVDc #1	8.22	7.84	0.36	4.36
PNVP-b-PVDc #2	5.64	5.64	0.41	3.10
PNVP-b-PVDc #3	6.56	6.30	0.59	4.78
PNVP-b-PVDc #4	5.01	5.17	1.07	3.05
PNVP-b-PVSt #1	3.51	3.52	0.59	3.41
PNVP-b-PVSt #2	1.97	2.11	1.60	2.82
PNVP-b-PVSt #3	4.60	4.62	0.30	5.14
PNVP-b-PVSt #4	8.00	7.46	1.20	3.83

## Data Availability

The original contributions presented in this study are included in the article. Further inquiries can be directed to the corresponding author.
